# Clinical Features and Computed Tomography Radiomics-Based Model for Predicting Pancreatic Ductal Adenocarcinoma and Focal Mass-Forming Pancreatitis

**DOI:** 10.1177/15330338231180792

**Published:** 2023-06-07

**Authors:** Yingjian Ye, Junyan Zhang, Ping Song, Ping Qin, Yan Hu, Peng An, Xiumei Li, Yong Lin, Jinsong Wang, Guoyan Feng

**Affiliations:** 1 Department of Radiology, 584878 Xiangyang No. 1 People's Hospital, Hubei University of Medicine, Xiangyang, China; 2 Department of Infectious Disease and Gastroenterology, Xiangyang Central Hospital, Affiliated Hospital of Hubei University of Arts and Science, Xiangyang, Hubei, China; 3 Department of Pharmacy and Laboratory, Xiangyang No. 1 People's Hospital, Hubei University of Medicine, Xiangyang, China; 4 Depatment of Radiology, Hubei Clinical Research Center of Parkinson's Disease, Xiangyang Key Laboratory of Movement Disorders, Xiangyang No. 1 People's Hospital, Hubei University of Medicine, Xiangyang, Hubei Province, P.R. China; 5 Department of Oncology, Xiangyang No. 1 People's Hospital, Hubei University of Medicine, Xiangyang, China; 6 Department of Internal Medicine, Xiangyang No. 1 People's Hospital, Hubei University of Medicine, Xiangyang, China

**Keywords:** mass-forming pancreatitis, pancreatic ductal adenocarcinoma, radiomics, prediction model, training set, test set, CT, ROC analysis, decision curve analysis, clinical decision-making

## Abstract

**Objective:** To establish a predictive model distinguishing focal mass-forming pancreatitis (FMFP) from pancreatic ductal adenocarcinoma (PDAC) based on computed tomography (CT) radiomics and clinical data. **Methods:** A total of 78 FMFP patients (FMFP group) and 120 PDAC patients (PDAC group) who were admitted to Xiangyang No.1 People's Hospital and Xiangyang Central Hospital from February 2012 to May 2021 and were pathologically diagnosed were included in this study, and were input to set up the training set and test set at a ratio of 7:3. The 3Dslicer software was used to extract the radiomic features and radiomic scores (Radscores) of the 2 groups, and the clinical data (age, gender, etc), CT imaging features (lesion location, size, enhancement degree, vascular wrapping, etc) and CT radiomic features of the 2 groups were compared. Logistic regression was used to screen the independent risk factors of the 2 groups, and multiple prediction models (clinical imaging model, radiomics model, and combined model) were established. Then the receiver operating characteristic (ROC) analysis and decision curve analysis (DCA) were conducted to compare the prediction performance and net benefit of the models. **Results:** The multivariate logistic regression results indicated that dilation of the main pancreatic duct, vascular wrapping, Radscore1 and Radscore2 were independent influencing factors for distinguishing FMFP from PDAC. In the training set, the combined model showed the best predictive performance (area under the ROC curve [AUC] 0.857, 95% CI [0.787-0.910]), significantly higher than the clinical imaging model (AUC 0.650, 95% CI [0.565–0.729]) and the radiomics model (AUC 0.812, 95% CI [0.759–0.890]). DCA confirmed that the combined model had the highest net benefit. These results were further validated by the test set. **Conclusion:** The combined model based on clinical–CT radiomics data can effectively identify FMFP and PDAC, providing a reference for clinical decision-making.

## Introduction

Mass-forming pancreatitis (MFP) is a special type of chronic pancreatitis mediated by abnormal digestive enzymes in the pancreas, alcohol abuse, or autoimmune reactions, and can be divided into diffuse and focal types morphologically.^1,2^ Diffuse MFP is often accompanied by lots of inflammatory exudates and various positive serum markers, but without local atrophy of the pancreas generally, which is easy to differentiate from tumor. Still, focal MFP (FMFP) and pancreatic ductal adenocarcinoma (PDAC) usually have similar clinical symptoms, laboratory findings and imaging performance, both are difficult to distinguish.^3,4^ Multimodal imaging signs and clinical manifestations of FMFP and PDAC often overlap, and early preoperative differential diagnosis is difficult. The treatment methods and prognosis of the 2 diseases are different. PDAC is a highly malignant tumor of the digestive system, and its neural infiltration and hematogenous metastasis seriously affect the life of patients.^5,6^ Whereas FMFP is an inflammatory disease of the pancreas, and 80% of patients may recover after medical or interventional therapy and diet control. At present early radical resection is an effective treatment for PDAC. There are many cases that misdiagnose FMFP as malignant tumor and take unnecessary surgery or misdiagnose PDAC as FMFP and delay the best operation time. Therefore, preoperative early differential diagnosis is the key to effectively implement accurate treatment and improve the prognosis of FMFP/PDAC patients. However, currently, in China, in order to avoid missed diagnosis of PDAC and medical disputes, at least 25% of FMFP patients have undergone unnecessary surgical resection due to high suspicion of pancreatic cancer. It increased financial burden and psychological trauma of patients, and may cause bowel adhesion, pancreatic fistula and other serious sequelae.^7,8^ Therefore, noninvasive and accurate preoperative identification of FMFP and PDAC is crucial. Enhanced computed tomography (CT) is the most widely used and the most important imaging method for differential diagnosis of PDAC and FMFP, but its accuracy depends on the clinical experience and subjective judgment of radiologists; the gold standard for distinguishing the 2 diseases mainly depends on puncture biopsy, which is not only invasive and has false negative, but also often may lead to problems such as tumor implantation and pancreatic leakage. As a new noninvasive auxiliary diagnosis technology, radiomics can extract a large number of image texture features that cannot be observed by the naked eye from traditional image images, and transform them into texture data information that can be mined and quantified and have important diagnostic and therapeutic value. At present, it is known that CT/magnetic resonance radiomics has achieved good research results in the differential diagnosis of brain tumors, breast tumors, pancreatic tumors and other diseases, but its report on the differential diagnosis of FMFP and PDAC is less, and it is generally a single-center retrospective study with limited sample size, which is inevitably biased.^9,10^ The purpose of this study is to explore the value of contrast-enhanced CT radiomics model in the differential diagnosis of FMFP and PDAC using radiomics, in order to provide a new and feasible scientific method for noninvasive differential diagnosis of the 2 diseases before operation, and to provide help for clinical decision-making and personalized treatment. Moreover, our team aimed to establish a simple predictive model based on clinical features-CT radiomics to identify FMFP and PDAC and achieved good results.

## Materials and Methods

### Case Data

**Figure 1.**
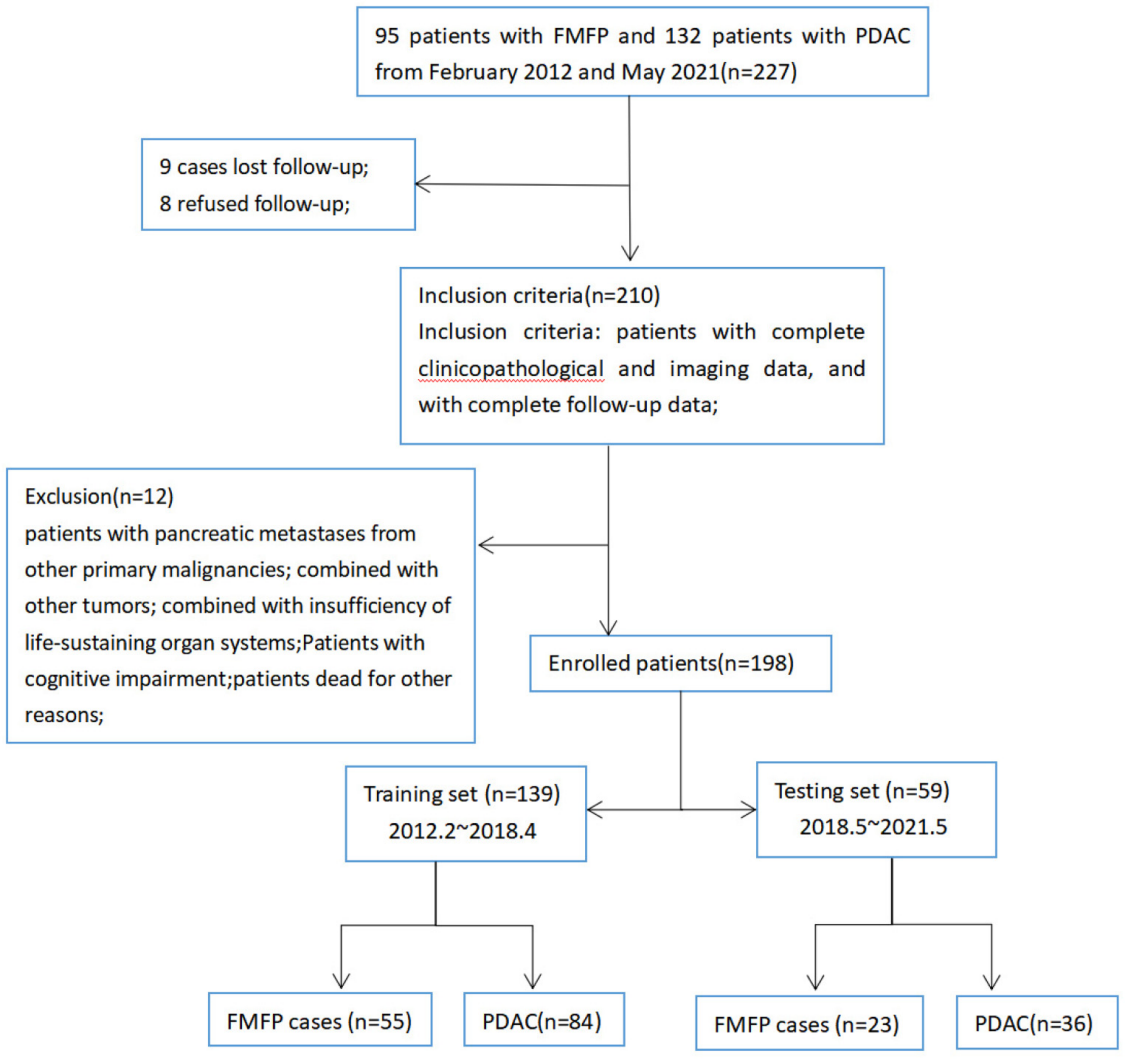
The simplified inclusion and exclusion criteria for patient enrollment in the present study.

We retrospectively randomly analyzed the clinical and radiographic data of 227 patients pathologically confirmed with FMFP or PDAC at XX Hospital (A) and XX Hospital (B) from February 2012 and May 2021, and then which were initially included in this study based on the pathological results (endoscopic ultrasonography-guided fine-needle aspiration biopsy or postoperative pathology), included 95 patients with FMFP (A:54, B:41) and 132 patients with PDAC (A:81, B:51). Inclusion criteria: patients with complete clinicopathological and imaging data, and with complete follow-up data. Exclusion criteria: patients with pancreatic metastases from other primary malignancies; combined with other tumors; combined with insufficiency of life-sustaining organ systems.^11^ Finally, a total of 78 FMFP patients (FMFP group) and 120 PDAC patients (PDAC group) were included. The XX Hospital includes 43 cases of FMFP and 72 cases of PDAC; the XX Hospital includes 35 cases of FMFP and 48 cases of PDAC. Our team stressed that the purpose of selecting a 2-center study is to reduce bias and expand the sample size of the study. Clinical data collected in this study includes: age, gender, body mass index, lesion size, lesion location, pancreatic tail atrophy, dilation of the common bile duct, dilation of the main pancreatic duct, acute obstructive pancreatitis, degree of enhancement, morphological changes of pancreas, pancreatic cyst, vascular wrapping, neural invasion, adjacent lymph node swelling, peripancreatic fluid, CA19-9, IgG4, and serum amylase. Our team divided 198 enrolled cases into training sets and test sets at a ratio of 7:3 (corresponding to the time node of May 2018); the training set (*n*  =  139) includes 55 FMFPs and 84 PDACs, and the test set (*n*  =  59) includes 23 FMFPs and 36 PDACs (Figure 1).

This is an exploratory, 2-center, and retrospective study. This study was approved by the XX Hospital and XX Hospital Institutional Review (Issue No. XX, No. XX). conducted in accordance with the Declaration of Helsinki. Written informed consent was obtained from individual or guardian participants. The reporting of this study conforms to Strengthening the Reporting of Observational Studies in Epidemiology (STROBE) guidelines.^11^ I have de-identified patient details such that the identity of any person may not be ascertained in any way.

### CT Scanning

The 64-slice CT scanner (Definition AS, Siemens Healthineers, Germany) and the spiral CT scanner (Aquilion 64, Toshiba Medical Systems Corporation, Japan) were used. CT scanning parameters were set as tube voltage 120 kV, tube current 160 mA, collimator width 140 mm  ×  0.5 mm, matrix size 400  ×  400, and slice thickness 1 mm. A scan extended from diaphragm to the lower border of the kidney. Enhanced scanning was performed while injecting 90–100 mL contrast agent iopromide (360 mg I/mL, Borui Biomedical, China) using a high-pressure syringe, at a flow rate of 6.0–6.5 mL/s. The arterial phase, portal venous phase and delayed phase images were obtained respective 20–30, 60–80, and 100–130 s after the injection.^12^ Our team emphasize that the CT series equipment and scanning parameters of this double-center retrospective study are the same, which is more standardized and can reduce bias.

### Image Analysis

**Figure 2.**
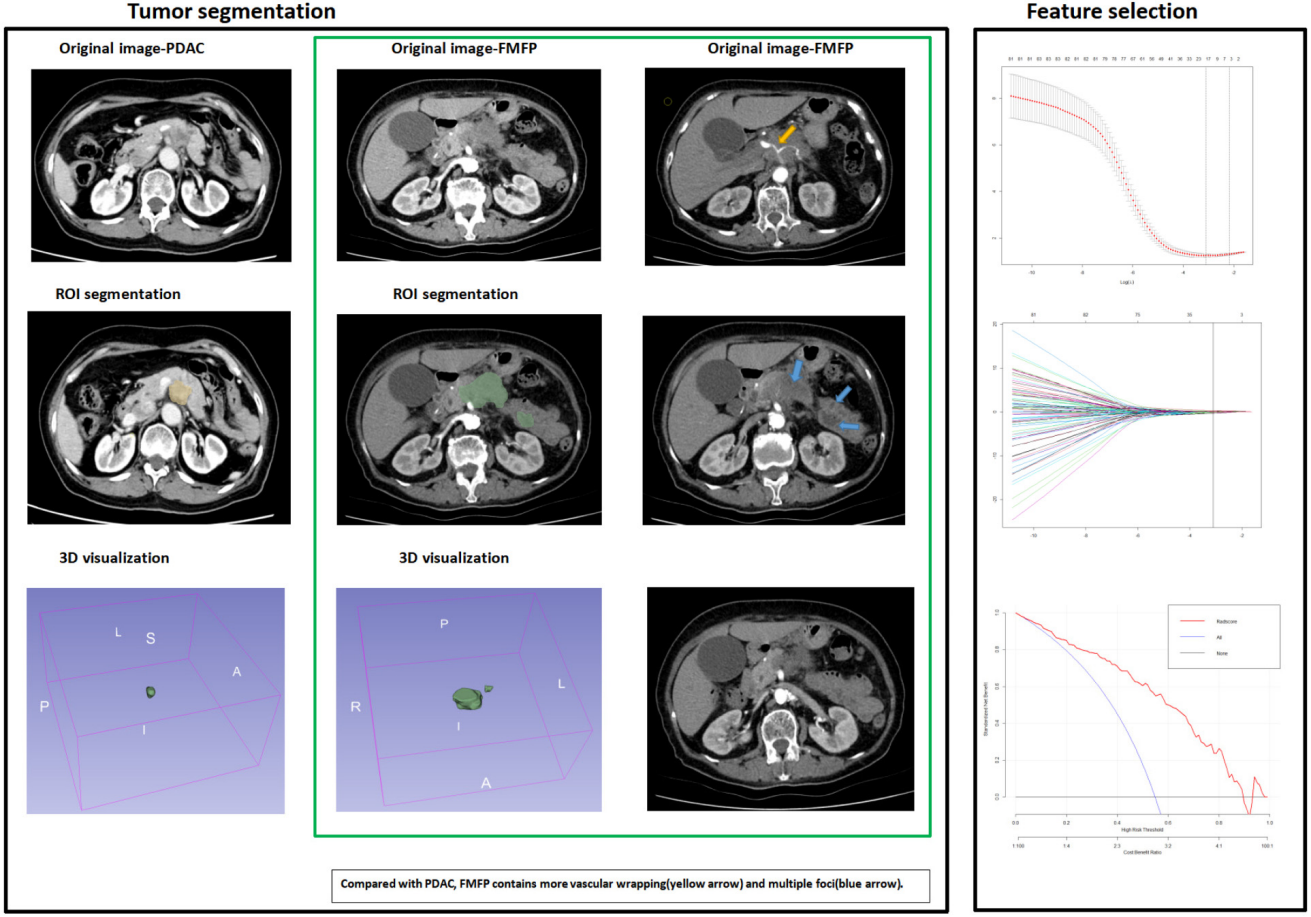
Schematic diagram of the simple process of CT radiomics extraction and the imaging histology circling the region sites associated with FMFP and PDAC in this study. We found that compared with PDAC, FMFP contains more vascular wrapping (yellow arrow) and multiple foci (blue arrow).

Abbreviations: CT, computed tomography; FMFP, focal mass-forming pancreatitis; PDAC, pancreatic ductal adenocarcinoma.

The results were independently reviewed by 2 senior specialists who had over 10 years of experiences in pancreas-related disease diagnosis. The indicators included lesion location, size, and degree of enhancement, pancreatic tail atrophy, dilation of the main pancreatic duct (upstream main pancreatic duct diameter >5 mm), dilation of the common bile duct (diameter >8 mm), cysts (retention cysts and pseudocysts), acute obstructive pancreatitis, vascular and neural invasion. CT radiomic texture extraction: in this study, 3D slicer (version 4.31) image segmentation software was used to delineate the pancreatic mass, and then texture analysis and data extraction were performed. After determining the candidate texture data such as glcm, glszm and shape, R (version 4.1.3) was then used to perform Lasso regression on the candidate texture data to extract effective texture data and generate radiomic scores (Radscores). Radscore1 was for arterial phase, Radscore2 was for venous phase, and Radscore3 was for delayed phase^13,14^ (Figure 2).

### Statistical Analysis

R (version 4.1.3, https://www.r-project.org/) was also used for statistical analysis. All continuous variables were tested for normal distribution and homogeneity of variance. The measurement data that conformed to the normal distribution were expressed as X  ±  S, and the *t*-test was used for comparison between groups. The measurement data that was not normally distributed was expressed as range, and the rank sum test was used for comparison between groups. The categorical variables were expressed as percentage of case (%), and the *X*^2^-test was used for comparison between groups. Logistic regression was conducted to analyze the differences in clinical data, general imaging features, and radiomics characteristics between groups. Then, the training set and test set at a ratio of 7:3 and several prediction models were established, and the area under the receiver operating characteristic curve (AUC) was calculated to evaluate and verify the diagnostic performance of the models. Then decision curve analysis (DCA) was performed to assess the clinical value of the models. *P* < .05 was considered statistically significant.^14^

## Results

### Comparison of Clinical and CT Imaging Features of 2 Groups of Patients

There were statistically significant differences in the dilation of the main pancreatic duct, tumor enhancement, vascular wrapping, carbohydrate antigen 19-9 (CA19-9), Radscore1, and Radscore2 between the 2 groups (*P* < .05), and there was no statistical difference in the other characteristics between these two groups (Tables 1 and 2).

**Table 1.** Logistic Regression Analysis Results of Clinical Imaging Model Based on Clinical/CT Characteristics for Predicting the FMFP and PDAC, *P < .05.

Clinical imaging model	Univariate analysis		Multivariate analysis	
Factors	*P*	Hazard ratio	*P*	Hazard ratio
Age	.14	1.02 (0.99–1.05)		
Gender	.54	1.20 (0.67–2.14)		
BMI	.43	0.96 (0.88–1.05)		
Lesion size	.13	0.98 (0.96–1.01)		
Lesion location	.68	0.93 (0.64–1.33)		
Pancreatic tail atrophy	.05	1.75 (0.98–3.11)		
Dilation of the common bile duct	.05	1.13 (0.98–1.29)		
Dilation of the main pancreatic duct	.03*	1.12 (1.01–1.24)		
Acute obstructive pancreatitis	.98	1.01 (0.56–1.78)		
Degree of enhancement	.04*	0.75 (0.57–0.98)		
Morphological changes in the pancreas	.72	1.11 (0.63–1.96)		
Pancreatic cyst	.45	1.24 (0.71–2.20)		
Vascular wrapping	.03*	0.53 (0.29–0.95)	.04*	0.53 (0.29–0.96)
Neural invasion	.25	0.71 (0.41–1.26)		
Adjacent lymph node swelling	.90	1.04 (0.59–1.83)		
Peripancreatic fluid	.74	1.10 (0.62–1.96)		
CA19-9	.03*	1.01 (1.00–1.02)		
IgG4	.12	0.99 (0.98–1.01)		
Serum amylase	.59	0.99 (0.99–1.01)		

Abbreviations: CT, computed tomography; FMFP, focal mass-forming pancreatitis; PDAC, pancreatic ductal adenocarcinoma; BMI, body mass index; CA19-9, carbohydrate antigen 19-9; IgG4, immunoglobulin G4.

**Table 2.** Logistic Regression Analysis Results of CT Radiomics Model for Predicting the FMFP and PDAC, *P < .05.

CT radiomics model	Univariate analysis		Multivariate analysis	
Factors	*P*	Hazard ratio	*P*	Hazard ratio
Radscore1	.01*	2.63 (1.93–3.58)	.01*	2.70 (1.95–3.75)
Radscore2	.01*	3.48 (1.91–6.34)	.01*	3.72 (1.87–7.39)
Radscore3	>.05	/		

Abbreviations: CT, computed tomography; FMFP, focal mass-forming pancreatitis; PDAC, pancreatic ductal adenocarcinoma; 2D, two-dimensional; MCC, Mathews correlation coefficient; Idmn, inverse difference moment normalized.

Radscore1  =  0.01412537 × maximum 2D diameter row  +  0.28366131 × gray level nonuniformity…88−0.00929523 × median…304−0.10318302 × small dependence low gray level emphasis…350−0.06920060 × contrast…479  +  0.36619653 × correlation…783  +  0.46126595.

Radscore2 = −0.148315597 × elongation−0.151533739 × Idmn…45  +  0.045966051 × skewness…123−0.143096467 × correlation…132−0.148606784 × large area high gray level emphasis…186−0.210068367 × small dependence low gray level emphasis…257−0.015909102 × mean…396  +  0.189269916 × correlation…411  +  0.041582512 × MCC…425−0.363134903 × gray level nonuniformity…460−0.279938588 × kurtosis…486−0.049643443 × Large area high gray level emphasis…558  +  0.006115149 × zone entropy…566  +  0.008949694 × gray level nonuniformity…646−0.017263353 × skewness…681−0.054561865 × correlation…690−0.198404207 × contrast…758−0.004054825 × kurtosis…765−0.175972963 × median…769−0.107852238 × minimum…770  +  0.07818119 × small dependence low gray level emphasis…815  +  0.485781005.

Radscore3 = −0.19747530 × median…211  +  0.10668183 × inverse variance…421  +  0.04693493 × run variance…735  +  0.43011272.

### Establishment of Predictive Models and Diagnostic Performance Validation

**Figure 3.**
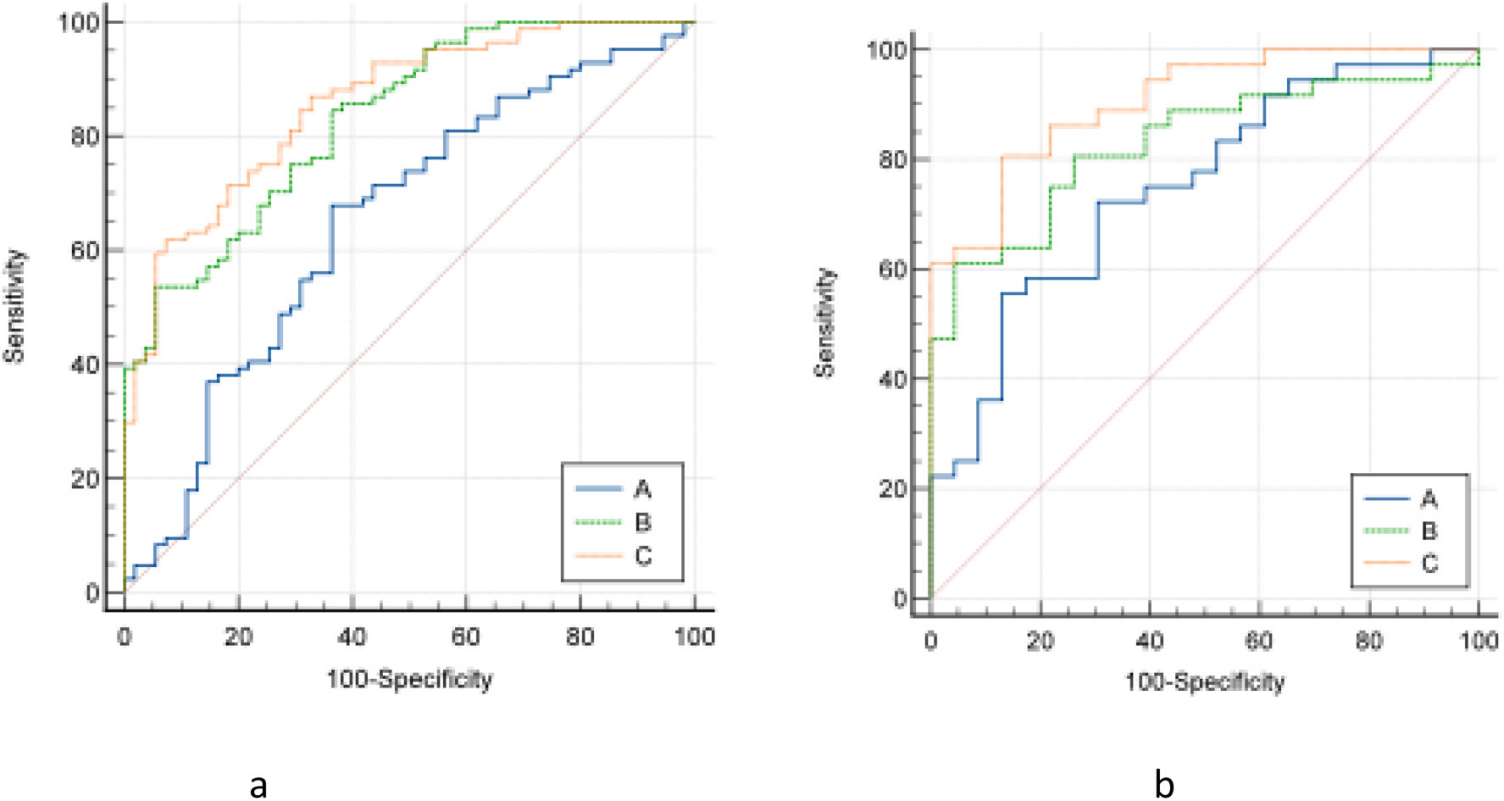
Delong nonparametric curves of the training set (a) and the test set (b). The area under the ROC curve of the combined model of the two groups is the largest; clinical imaging model (A); CT radiomics model (B); and combined model (C).

Abbreviations: ROC, receiver operating characteristic; CT, computed tomography.

**Figure 4.**
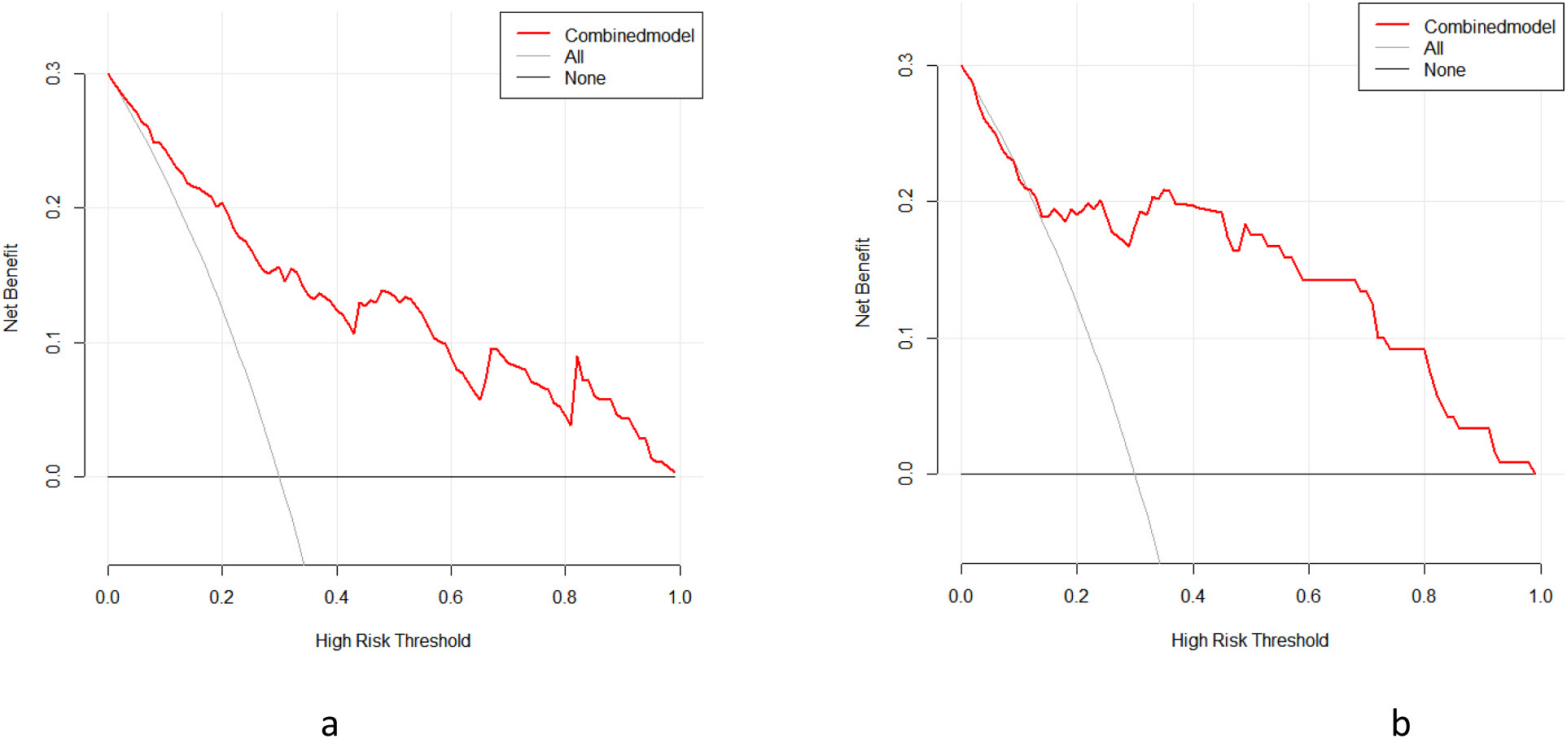
The higher net benefits of the combined model were confirmed in the 2 groups by decision curve analysis (DCA) of the training set (a) and test set (b) using R software.

**Figure 5.**
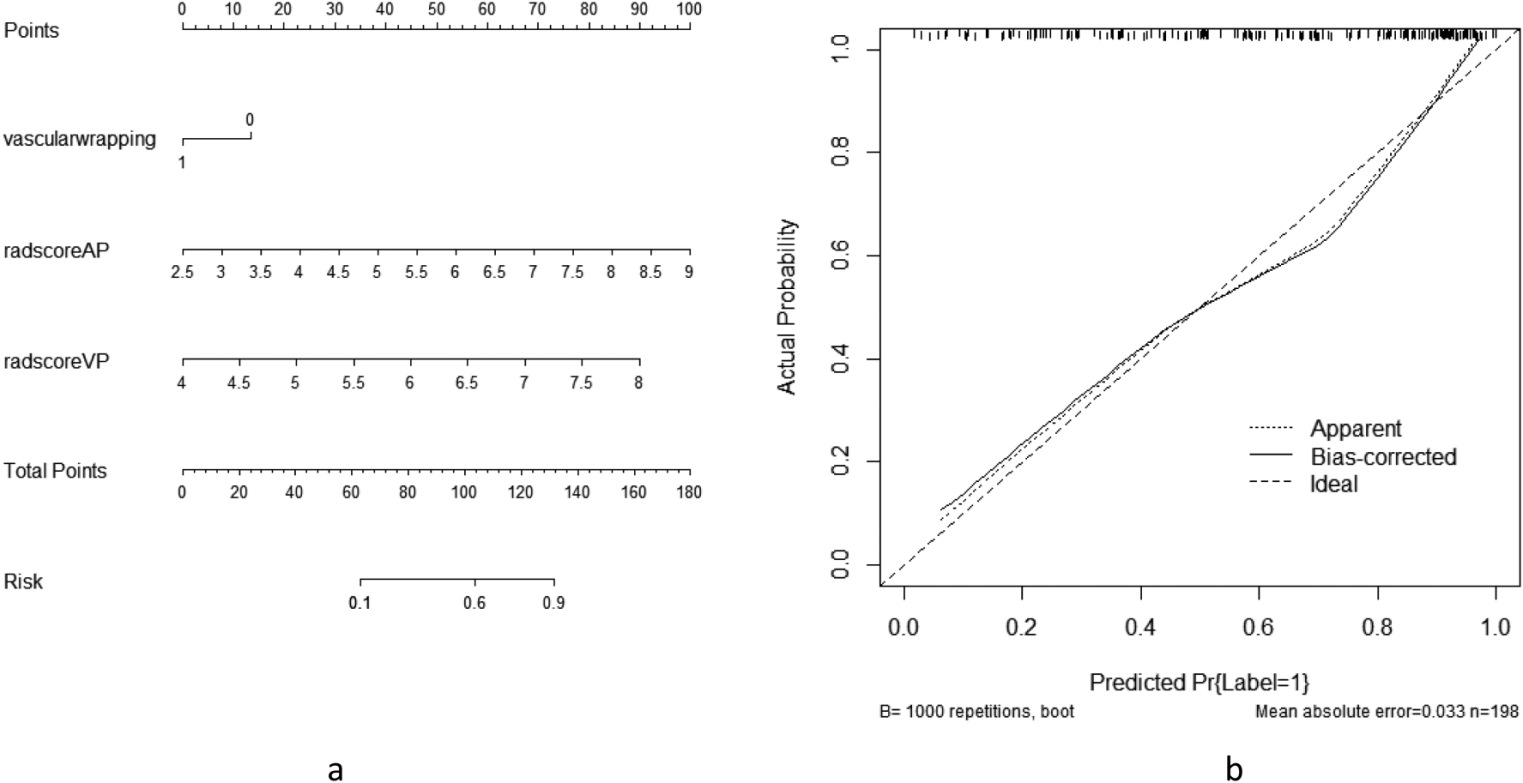
The nomogram prediction tool based on the risk factors of the combined model was used clinically (a, nomogram; b, calibration curve).

According to the results of univariate logistic regression analysis, 3 models, a clinical imaging model (included dilation of the main pancreatic duct, tumor enhancement, vascular wrapping, and CA19-9), a radiomics model (Radscore1, and Radscore2), and a combined model (dilation of the main pancreatic duct, tumor enhancement, vascular wrapping, CA19-9, Radscore1, and Radscore2) were established, respectively (Tables 2 and 3). In the training set, the combined model gave the best predictive performance (AUC 0.857, 95% CI [0.787–0.910]), significantly better than the clinical imaging model (AUC 0.650, 95% CI [0.565-0.729]) and the CT radiomics model (AUC 0.812, 95% CI [0.759-0.890]). In the test set, the combined model also gave the best predictive performance (AUC 0.906, 95% CI [0.801-0.966]), significantly better than the clinical imaging model (AUC 0.750, 95% CI [0.620-0.854]) and the radiomics model (AUC 0.820, 95% CI [0.709-0.915]). DCA confirmed high net benefits of the combined model in both sets. It indicated that the combined model had good diagnostic performance in the differential diagnosis of FMFP and PDAC (Figures 3 and 4). The nomogram generator tool and the calibration curves developed using the R software had been used in clinical settings (Figure 5).

**Table 3.** Logistic Regression Analysis Results of Combined Model Based on Valuable Factors Mentioned Above for Predicting the FMFP and PDAC, *P < .05.

Combined model	Univariate analysis		Multivariate analysis	
Factors	*P*	Hazard ratio	*P*	Hazard ratio
Dilation of the main pancreatic duct	.03*	1.12 (1.01–1.24)		
Degree of enhancement	.04*	0.75 (0.57–0.98)		
Vascular wrapping	.03*	0.53 (0.29–0.95)	.02*	0.43 (0.21–0.91)
CA19-9	.03*	1.01 (1.00–1.02)		
Radscore1	.01*	2.63 (1.93–3.58)	.01*	2.65 (1.89–3.72)
Radscore2	.01*	3.48 (1.91–6.34)	.01*	4.16 (1.99–8.55)

Abbreviations: FMFP, focal mass-forming pancreatitis; PDAC, pancreatic ductal adenocarcinoma; CA19-9, carbohydrate antigen 19-9.

## Discussion

With the soaring economy in China in recent years, issues such as environmental pollution, food safety, and eating disorder are getting severer, and the incidence of pancreatic tumors is rising in China, reaching 5.1 per 100,000. At the same time, the incidence of pancreatitis also reached 3%.^15,16^ MFP is a special type of chronic pancreatitis. Histologically, it can be divided into lymphoplasmacytic infiltration pancreatitis and idiopathic pancreatitis centered on the pancreatic duct. Morphologically, it can be divided into diffuse type and focal type. Diffuse MFP has a typical sausage-like appearance, clear borders, and pseudocapsule structure, making it easier to be distinguished from PDAC. Whereas both FMFP and PDAC are manifested as focal pancreatic mass and pancreatic duct dilation. The identification of these 2 remains challenging now. And the treatment and prognosis of these 2 are completely different. In the past, to play safe, most Chinese FMFP patients chose surgical resection. However, the postoperative intensive care unit care was difficult, and the wound healed slowly, having risks such as pancreatic leak and perforation. All of these resulted in huge economic pressure and psychological burden on the patient.^17–19^ Everyone seems to think that, in clinic practice, experienced clinicians can identify FMFP and PDAC according to relevant tumor markers, inflammatory indicators, CT, magnetic resonance imaging (MRI), and so on. However, many previous research reports and this study have found that the 2 have many similarities. It is difficult to distinguish the 2 by simply relying on medical history and biochemical indicators. Although CT/MRI is easy to identify the imaging features of both pancreatic masses, it is difficult to accurately confirm the FMFP with large volume and blood vessel wrapping before operation. Pancreatic surgeons are also caught in a dilemma and often misdiagnosed as PDAC, which led to unnecessary surgery and endoscopic ultrasound guided biopsy. Therefore, the pretreatment identification of FMFP and PDAC is particularly important, and the CT radiomics-based combination model developed in this study has achieved good results and brought opportunities to us.

In the past, the diagnosis of FMFP was based on the Mayo Clinic HISORt criteria (histology findings, imaging, serology, other organ involvement, and response to steroid therapy) from the United States, but each examination method has certain limitations. For example, it is still controversial about the roles of dilation of the common bile duct, dilation of the main pancreatic duct, and the sensitivity and specificity of CA19-9 or IgG4 in identifying FMFP and PDAC. Endoscopic ultrasonography-guided fine-needle aspiration (EUS-FNA) is the gold standard for the preoperative diagnosis of pancreatic mass, but it is invasive, time-consuming, expensive, and highly heterogeneous among different doctors. It is reported that the confirmed diagnosis rate of FMFP by EUS-FNA was only 68.2%. In addition, EUS-FNA cannot provide a satisfactory diagnosis rate even in the study of large tissue samples. Therefore, CT and MRI have become the most used noninvasive diagnostic methods to identify FMFP. It has been reported that the sign of pancreatic duct perforation and significant enhancement in the arterial phase on CT or EUS-FNA were specific signs supporting the diagnosis of FMFP.^19–21^ Actually, this study found that 33.6% of FMFP patients had uniform enhancement and 35.2% had significant enhancement in the venous phase, and 28.5% of FMFP patients had strong enhancement in the delayed phase. These findings have important diagnostic value. While PDAC was a hypovascular tumor, and 46.2% patients showed uniform enhancement, 32.2% patients had mild enhancement in venous phase; and 7.9% showed significant enhancement. The differences in enhancement and pattern between the 2 groups were statistically significant, which is also a good complement to the previous conclusion.

In addition to the enhancement mode, this study also found that widening of the main pancreatic duct, vascular wrapping, CA19-9, Radscore1, and Radscore2 were the influencing factors to distinguish the 2. The lesions in PDAC patients are often located in the head and body of the pancreas, and the masses often infiltrate the pancreatic duct to cause obstruction, so the atrophy of the pancreatic tail and the widening of the pancreatic bile duct are common; CA19-9 is a routine positive indicator of gastrointestinal tumors. It is reported that CA19-9 is also elevated in 60%–70% sensitive PDAC. However, FMFP often presents vascular wrapping, because FMFP volume is often larger than PDAC and the symptom development is not obvious, and FMFP is often a benign disease. Therefore, FMFP often surrounds blood vessels and presents inflammatory exudation, but does not infiltrate and block blood vessels, which is a classic imaging manifestation of inflammatory lesions. Radiomics is an emerging auxiliary diagnosis technology developed in recent years. It can mathematically analyze the distribution of pixels in medical images, obtain quantitative parameters of a series of lesions with high throughput, and fully present the most essential characteristics of the underlying medical images.^22,23^ In this study, a clinical–CT radiomics model was established for the identification of FMFP and PDAC. The present model contained 3 CT image-based radiomics features and performed well in identifying these 2 diseases, clearly complementing traditional clinical data. Thin-slice CT imaging have been reported to distinguish tumors better than thick-slice CT imaging. Therefore, our extracted texture-omics parameters based on thin-slice CT were remarkably more valuable. Radscore1 and Radscore2 are the comprehensive scores representing the arterial phase and venous enhanced imaging histology of pancreatic masses and represent the changes of the imaging underlying texture parameters of the lesions, so they have important diagnostic values.^24,25^ In this study, the effective indicators screened by multivariate analysis were further subjected to modeling analysis, and finally a series of independent risk factors such as dilation of the common bile duct, vascular wrapping, Radscore1, and Radscore2 were extracted. The subsequent DeLong curves confirmed that the combined model had higher predictive power, and DCA also confirmed its high net clinical benefit. Our study contributes to the clinical management of FMFP and PDAC and avoids unnecessary surgery and invasive treatments.

## Limitation

Since this was a 2-center study with a small sample size, we did not calculate sample size using random sampling, whole group sampling or other statistical methods. Besides, only the conventional logistic regression analysis was employed, which may miss some valuable data and result in bias. In the future, multicenter studies, machine learning and artificial intelligence methods would be introduced for research and validation.^26^

## Conclusions

In conclusion, it is feasible to establish a model based on clinical radiomics data to identify FMFP and PDAC, which may help to improve their clinical treatment decisions and prognosis.

## Abbreviations

AUC: area under the receiver operating characteristic curve
CT: computed tomography
DCA: decision curve analysis
EUS-FNA: endoscopic ultrasonography-guided fine-needle aspiration
MFP: mass-forming pancreatitis
FMFP: focal MFP
MRI: magnetic resonance imaging
PDAC: pancreatic ductal adenocarcinoma
ROC: receiver operating characteristic.
